# AnnexinA7 promotes epithelial–mesenchymal transition by interacting with Sorcin and contributes to aggressiveness in hepatocellular carcinoma

**DOI:** 10.1038/s41419-021-04287-2

**Published:** 2021-10-29

**Authors:** Fei Ling, Huan Zhang, Yunliang Sun, Jinyi Meng, Jaceline Gislaine Pires Sanches, He Huang, Qingqing Zhang, Xiao Yu, Bo Wang, Li Hou, Jun Zhang

**Affiliations:** 1 Department of Pathology and Forensics, College of Basic Medical Sciences, Dalian Medical University, Dalian, 116044 China; 2 Department of Pathology, Dalian Municipal Central Hospital affiliated with Dalian Medical University, Dalian, 116033 China

**Keywords:** Medical research, Genetics research

## Abstract

Hepatocellular carcinoma (HCC) is one of the most common cancers worldwide, and metastasis is the major cause of the high mortality of HCC. In this study, we identified that AnnexinA7 (ANXA7) and Sorcin (SRI) are overexpressed and interacting proteins in HCC tissues and cells. In vitro functional investigations revealed that the interaction between ANXA7 and SRI regulated epithelial–mesenchymal transition (EMT), and then affected migration, invasion, and proliferation in HCC cells. Furthermore overexpression/knockdown of ANXA7 was remarkably effective in promoting/inhibiting tumorigenicity and EMT in vivo. Altogether, our study unveiled a mechanism that ANXA7 promotes EMT by interacting with SRI and further contributes to the aggressiveness in HCC, which provides a novel potential therapeutic target for preventing recurrence and metastasis in HCC.

## Introduction

Hepatocellular carcinoma (HCC) is a highly aggressive malignant tumor, which has become one of the leading cancer-associated death in many countries currently. Evidence suggests that the incidence of HCC is increasing. Despite significant developments in the diagnosis and treatment, the overall survival of patients with HCC remains low due to distant metastasis. The precise molecular mechanism is complex and still unclear. Epithelial–mesenchymal transition (EMT) is a cellular program in which epithelial cells switch to a mesenchymal phenotype, not only crucial for embryogenesis, organogenesis, and chronic inflammation but also affects cancer metastasis [1–6]. The EMT process has been described as the crucial step in the malignant progression and dissemination of the carcinoma cells [7]. Identification of the molecular mechanisms underlying EMT-induced progression of HCC may be a potential new therapeutic target for HCC treatment.

AnnexinA7 (ANXA7) is a Ca^2+^ and phospholipid-binding protein, existing in a 47 and 51-kDa isoform. It can inhibit the activity of phospholipase A in cells, participating in endocytosis and exocytosis, cell proliferation, differentiation, apoptosis, and signal transduction [8, 9]. In addition, it has an important role in the inflammatory response, anticoagulation, and tumor metastasis. ANXA7 has different roles in different tumors [10–13]. We have previously identified ANXA7 as an important gene/protein in HCC. Nevertheless, the mechanisms of ANXA7 in HCC are still unclear and further research is needed.

Soluble Resistance-related Calcium-binding protein (Sorcin, SRI) belongs to the PEF (Penta-EF-hand) protein family that contains five EF-hand motifs that associate in the calcium-dependent manner [14, 15]. It is widely distributed among mammalian tissues such as the heart, kidney, muscle, brain, adrenal medulla, and overexpressed in many tumor cells [16–22]. SRI has a crucial role in the occurrence development and prognosis of tumors. SRI is also highly expressed in different drugs resistant cell lines, and its overexpression confers multiple drug resistance in a variety of human cancer cell lines [23–25]. SRI undergoes conformational changes when it binds to Ca^2+^, enabling it to interact with target proteins [26–30]. It has also been reported that SRI promotes the progress of EMT in colorectal cancer [31]. However, the mechanisms of SRI in metastasis of HCC are still lacking [32–34].

Increasing evidence suggests that protein often does not function as a single substance but rather as team players in a dynamic network and that protein–protein interaction (PPI) is crucial in many biological processes [35–37]. It is reported that ANXA7 interacts with SRI in a Ca^2+^-dependent manner. The first 31 amino acids in the ANXA7 sequence comprise several glycine residues which give rise to three GYPP structural motifs that interact with the GGYY and the GYGG sequences in the SRI N-terminal domain [37–39]. Therefore, we hypothesized that the interaction between ANXA7 and SRI might promote the progress of EMT, and then contribute to the metastasis of HCC.

## Materials and methods

### Bioinformatics analysis

Gene data about HCC were from The Cancer Genome Atlas (TCGA). The expression levels of ANXA7 and SRI were analyzed in normal liver tissues and HCC tissues. Each point represents an independent sample in the box plot. The potential PPI possibility was predicted by the STRING database.

### Cell line culture

Human hepatocytes (HL-7702) and HCC cell lines (Huh-7 and Hep3B) were obtained from the Cell Bank of Chinese Academy of Sciences. HL-7702 cell was cultured in 90% RPMI1640 medium (Hyclone, Logan, UT, USA) supplemented with penicillin/streptomycin and 10% fetal bovine serum (Biowest, France). Huh-7 and Hep3B cells were cultured in Dulbecco’s Modified Eagle Medium (DMEM)/high glucose (Hyclone, USA) containing 10% FBS at 37 °C with 5% CO_2_.

### Recombinant lentivirus

Lentivirus vectors include upANXA7, shANXA7 (5′-GGATATGTAGAAAGTG GTTTG-3′), and negative control ANXA7. During the reverse verification process, upSRI, shSRI (5′-GCCCTGACAACAATGGGATTT-3′) and negative control SRI had been constructed. The lentivirus vectors were purchased from GenePharma Co. Ltd (Shanghai, China). The transfected cells were selected using puromycin (Solarbio, Beijing, China).

### Stable transfection of cells

Huh-7 and Hep3B cells were divided into four groups: (a) unmanipulated Huh-7 and Hep3B cells were used as the control; (b) lentivirus vectors of upANXA7 and upSRI transfected into cells respectively; (c) lentivirus vectors of shANXA7 and shSRI transfected into cells respectively; (d) lentivirus vectors of negative control ANXA7 and SRI transfected into cells, respectively.

Huh-7 and Hep3B cells (2 × 10^5^ cells/well) were plated into a six-well plate, cells in different groups were transfected with 5 μg lentivirus vectors and 1 μl Polybrene using GenePharma^TM^ recombinant lentivirus reagent according to the manufacturer’s protocol. Transfection efficiencies were observed with fluorescence microscopy at 48–72 h. For the selection of stably transfected cells, puromycin was introduced at a concentration of 2 μg/ml for 2 weeks.

### RNA extraction and real-time quantitative PCR analysis

Total RNA was isolated from transfected cells and tissues using Trizol reagent (Transgen, Beijing, China), following the manufacturer’s protocol. In total, 1 μg of total RNA was reverse-transcribed with the All-in-one First-Strand cDNA Synthesis SuperMiX kit (Transgen, Beijing, China). The qRT-PCR was analyzed with the iCycler™ Real-Time System and aSYBR Premix EX Tag Master mixture kit (Transgen, Beijing, China) according to the manufacturer’s instructions. The relative expression levels of mRNA were normalized to GAPDH expression. The primer sequences are as follows:

ANXA7-Forward: AAGGGTTTGGGACAGACGA.

ANXA7-Reverse: CATGAACAGCGCAAGGATTA.

SRI-Forward: TTTCCCGGACAAACTCAGGAT.

SRI- Reverse: GAAACCCATTGTGCCAGACAT.

GAPDH-Forward: AAATGGTGAAGGTCGGTGTGAAC.

GAPDH-Reverse: CAACAATCTCCACTTTGCCACTG.

### Western blotting

Cells and tissues were lysed in RIPA cell lysis buffer (KeyGENBioTECH Corp. Ltd, Beijing, China). Protein concentrations were estimated using the QuantiPro™ BCA Assay Kit (Sigma-Aldrich Co. LLC, USA). Total protein (50ug) was resolved by sodium dodecyl sulphate–polyacrylamide gel electrophoresis on 12% polyacrylamide SDS gels and transferred to polyvinylidene fluoride (PVDF) membranes (Merck KGaA, Darmstadt, Germany). The PVDF membranes were blocked with 5% skim milk TBST at room temperature, then incubated with the primary antibody solution at 4 °C overnight. The specific antibodies including ANXA7 (Abcam Cambridge, MA, USA), 1:500; SRI (Proteintech, Wuhan, China), 1:500; E-cadherin (Proteintech, Wuhan, China), 1:500; Cytokeratin (Proteintech, Wuhan, China), 1:500; N-cadherin (Proteintech, Wuhan, China), 1:500; Vimentin (Proteintech, Wuhan, China), 1:500; GAPDH (Proteintech, Wuhan, China) 1:1000. After 24 h, the membranes were then washed, incubated with anti-IgG secondary antibodies 1:10000 (LI-COR, USA) at 37 °C for 2 h. The target protein bands were detected using Odyssey infrared imaging.

### Immunofluorescence analysis

HCC cells of each group were grown to 1 × 10^5^ in 48-well plates and fixed with 100% methanol. Then, the cells were washed with PBS two or three times and blocked with immunofluorescence blocking solution (Cell Signaling, USA) for 2 h at 37 °C. All cells were treated with the primary antibodies (Proteintech, Wuhan, China) overnight at 4 °C: ANXA7, 1:100; SRI, 1:100; E-cadherin, 1:100; Cytokeratin, 1:100; N-cadherin, 1:100; Vimentin, 1:100. Afterwards, cells were incubated with fluorescently-labeled secondary antibody (Sigma, 1:200) for 1 h at 37 °C and stained the cell nuclei with DAPI (Beyotime, Shanghai, China). Images were visualized and captured with CKX41 Inverted Microscope (Olympus, Japan).

### co-Immunoprecipitation (co-IP) assay

HCC cells treated with IP lysis buffer. Cell lysates were incubated with primary antibodies to generate the immune complexes overnight at 4 °C, and then agarose beads were added. After incubation, the binding proteins were eluted from the beads. The experiment was performed as described in the manufacturer’s protocols (IP Kit, PTGLAB, USA). Finally, protein A/G agarose could be used to capture target immune complexes, and then elutes were submitted to the immunoblotting.

### Immunohistochemical analysis

Thirty specimens of HCC tissue were collected from surgical specimens at Dalian Municipal Central Hospital Affiliated with Dalian Medical University. Tumor tissue was paraffin-embedded then deparaffinized in xylene and rehydrated with ethanol, and then immunostained with corresponding antibodies. Endogenous peroxidase activity was blocked by 30 min with normal goat serum, and the slides were incubated with primary antibody solution at 4 °C overnight. The following day the slides were washed and incubated with the secondary antibody and then DAB staining. The analysis of IHC staining was done by Image J software.

### Cell proliferation assay

The Cell proliferation was investigated using CCK8 (Beyotime, Shanghai, China). In all, 5 × 10^3^ cells/ml were plated into a 96-well plate and 1 μl of CCK8 solution was added into each well at 0 h, 12 h, and 24 h. All assays were performed in triplicate. The OD value of the cells was measured at 450 nm using a multifunctional microplate reader (Thermo Fisher, USA).

### Cell invasion assay

The Cell invasion was investigated using a Transwell culture insert (8 μm pore size, Corning, Tewksbury, MA, USA) coated with Matrigel (BD, Bioscience, San Jose, CA, USA) by incubating at 37 °C for 1 h. A total of 1.5 × 10^5^ cells in each group were seeded serum-free in the upper chamber of the 24-well plates. DMEM/high-glucose medium containing 20% serum was added into the lower chamber. After 16–18 h of incubation, the culture insert was removed and cells that remained in the culture insert were washed off with PBS. The cells attached to the lower side of the culture insert were fixed in methanol and stained with Giemsa solution (Solarbio, Beijing, China) and counted by a microscope.

### Wound-healing assay

Cells in each group were incubated in six-well plates. The cell monolayer was wounded with a 200 μl micropipette tip when cellular density reached nearly 80%. Then PBS was used to wash the scratch area three consecutive times. Cells were then cultured in the serum-free DMEM/high-glucose medium and incubated for 12 h and 24 h. The wound areas were micrographed at 0 h, 12 h, and 24 h. All assays were performed in triplicate and the images were analyzed by Image J software.

### Mouse xenograft model

Six-eight-week-old BALB/c Nude mice (male and female), were purchased from Cavenslasales Company (Changzhou, China) and maintained in the animal experiment center according to the guidelines of the Institutional Animal Care and Use Committee of Dalian Medical University. All animal care and experiments were conducted in standard treatment protocols. BALB/C nude mice were divided randomly into four groups including (a) unmanipulated group; (b) upANXA7 group; (c) shANXA7 group; (d) negative control of ANXA7 group. Huh-7 cells (1.5 × 10^6^) in each group were injected into the neck and back of nude mice. After 4 weeks of inoculation, the tumors and metastatic lymph nodes were removed, then weighed and analyzed by HE staining, qRT-PCR, western blotting, and immunohistochemistry.

### Statistical analysis

The experimental data were statistically analyzed by using GraphPad Prism 8.0 and SPSS 17.0. Differences between the two groups were evaluated by Student’s *t* test. One-way ANOVA was used to perform multiple comparisons. Metastatic lymph node in vivo experiments was calculated by Chi-square test. The data were presented as mean ± SD and statistical significance was set at *p* < 0.05.

## Results

### ANXA7 and SRI are overexpressed in hepatocarcinoma cells and tissues, and there is a positive correlation between them

**Fig. 1:**
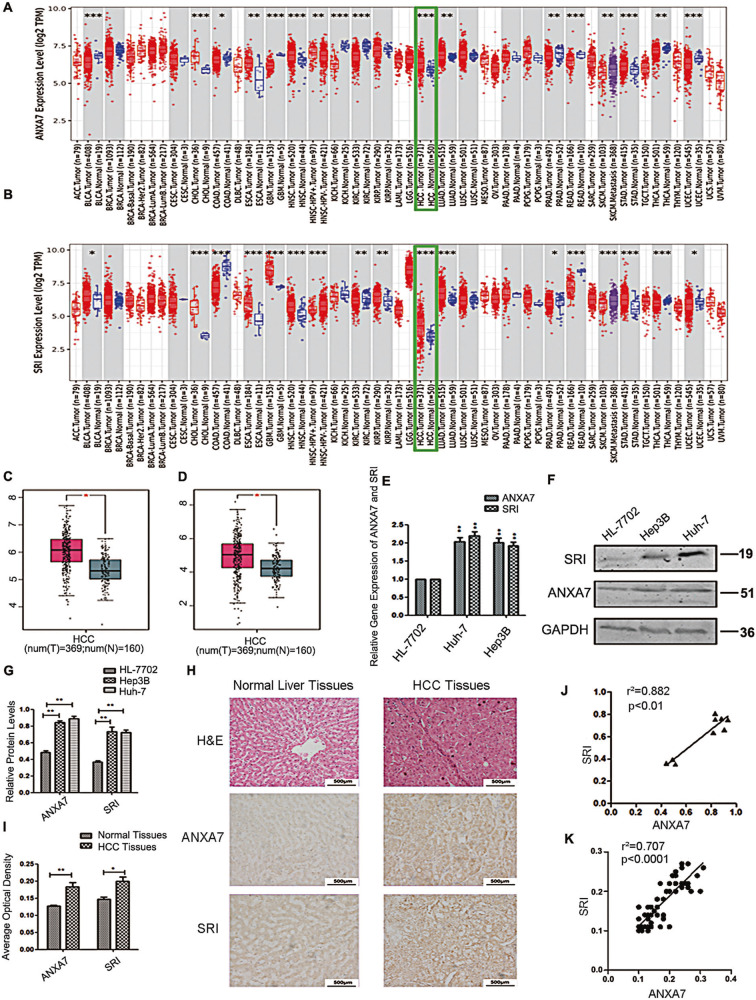
The expression of ANXA7 and SRI and the positive correlation between them in HCC. **A**, **B** The expression of ANXA7 and SRI in different cancers or specific cancer subtypes was analyzed through TIMER2. The expression of ANXA7 and SRI in HCC are higher (*n* = 371) than normal liver tissues (*n* = 50) (***p < 0.001). **C**, **D** The expression of ANXA7 is higher in HCC (*n* = 369) than in normal liver tissues (*n* = 160) from the TCGA database. The expression of SRI is also higher in HCC (*n* = 369) than in normal liver tissues (*n* = 160) from the TCGA database (^*^p < 0.05). **E**–**G** The expression of ANXA7 and SRI were higher in Huh-7/Hep3B cells than in normal human hepatocytes HL-7702 by qRT-PCR and western blotting. **H** Representative images of ANXA7 and SRI expression in normal human liver tissues and HCC tissues. **I** Quantification of the average means optical density for ANXA7 and SRI in normal liver tissues and HCC tissues (**p < 0.01, *p < 0.05). **J** ANXA7 expression level *p*ositively correlated with SRI expression in HCC cells (*r*^2^ = 0.882, *p* < 0.01). **K** ANXA7 expression level positively correlated with SRI expression in HCC tissues (*r*^2^ = 0.707, *p* < 0.01).

We applied the TIMER2 approach to analyze the expression status of ANXA7 and SRI in a panel of human cancers using the TCGA database and found the relative overexpression of ANXA7 and SRI in most human cancers (Fig. 1A, B). We further examined the TCGA to evaluate the expression of ANXA7 and SRI in HCC. The database analysis indicated that ANXA7 and SRI had a significantly higher expression in HCC samples (*n* = 369) than normal samples (*n* = 160) (Fig. 1C, D). To further confirm the overexpression of ANXA7 and SRI, we utilized a series of human hepatocarcinoma cells and tissues. The qRT-PCR, and western blotting results showed that the expression of ANXA7 and SRI in Huh-7 and Hep3B cells were higher than in normal human hepatocytes HL-7702 cells (Fig. 1E–G). Immunohistochemistry results further confirmed that the expression level of ANXA7 and SRI in HCC tissues was higher than that of normal liver tissues (Fig. 1H, I).

Furthermore, the inter-correlations of ANXA7 and SRI expression changes in hepatocarcinoma cells and tissues were analyzed. The result demonstrated a positive correlation between ANXA7 and SRI overexpression both in hepatocarcinoma cells (Fig. 1J) and tissues (Fig. 1K). These results indicated their high expression closely correlated in involving the development and progression of hepatocarcinoma.

### ANXA7 and SRI are interacting proteins

**Fig. 2:**
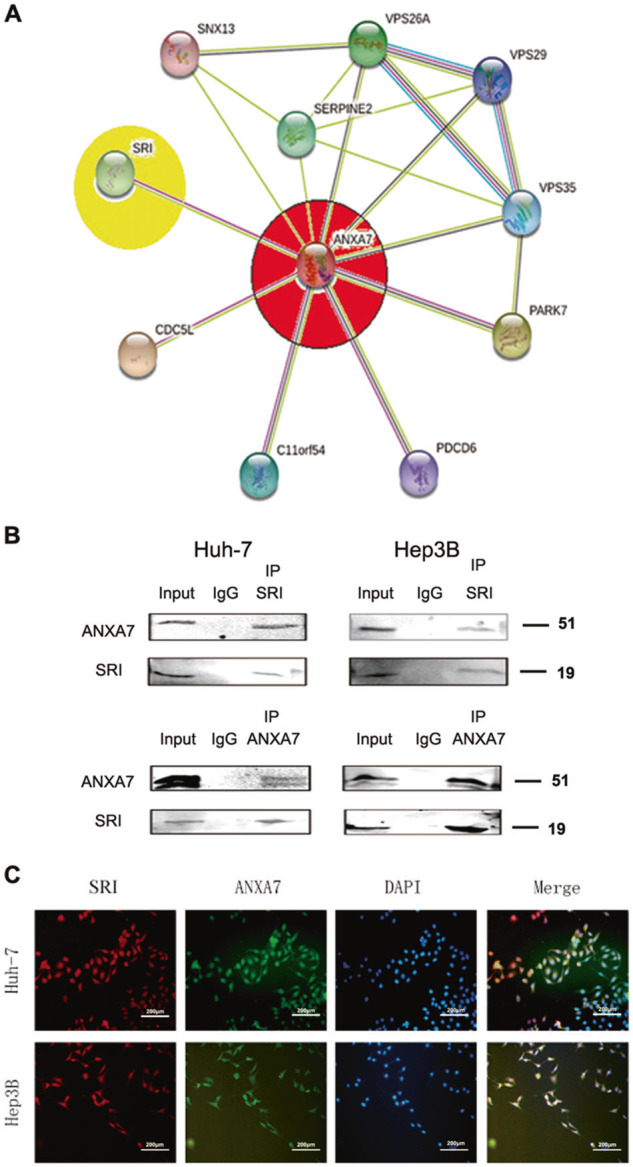
ANXA7 and SRI are interacting proteins. **A** There may be a potential interaction between ANXA7 and SRI, which was analyzed by the STRING database. **B** co-IP assay showed the interaction between ANXA7 and SRI in Huh-7/Hep3B cells. **C** Cellular immunofluorescence proved that ANXA7 and SRI are colocalized in the cytoplasm and cell membrane in Huh-7/Hep3B cells.

To analyze the protein interaction between ANXA7 and SRI, we assessed the STRING database. The PPI between ANXA7 and SRI from the STRING database demonstrated a potential interaction between them (Fig. 2A). To gain insights into ANXA7 and SRI function and regulation, we investigated whether they are interaction partners using co-IP assay and immunofluorescence staining. The co-IP assay demonstrated a direct interaction between ANXA7 and SRI in Huh-7 and Hep3B cells (Fig. 2B). Immunofluorescence staining was used to determine the subcellular localization, which revealed that both ANXA7 and SRI were colocalized in the cell membrane and cytoplasm in Huh-7 and Hep3B cells (Fig. 2C). Collectively, these data confirmed that ANXA7 and SRI are proteins that interact with each other.

### ANXA7 positively regulates the expression of SRI and promotes EMT

To investigate the effect of ANXA7 on SRI expression and EMT in HCC cells, lentivirus vectors were used to change the expression of ANXA7, and the transfection efficiency was up to 90% after 72 h. In addition, 2 weeks after selection by puromycin, the expression of ANXA7 in lentivirus infected Huh-7 and Hep3B cells was detected by qRT-PCR and western blotting. The stable cell lines (Huh-7 and Hep3B) upregulating and downregulating ANXA7 were established (Fig. S1A).

**Fig. 3:**
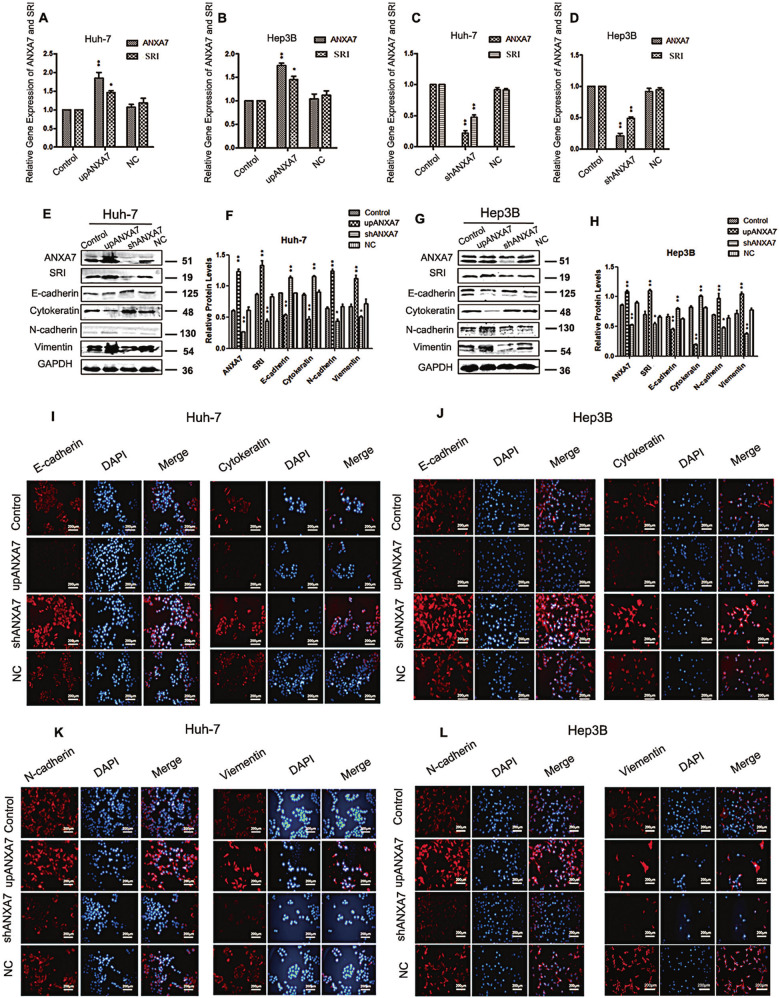
ANXA7 expression positively regulates SRI expression and affects EMT in HCC cells. **A**–**D** Upregulated ANXA7 increased SRI expression and downregulated ANXA7 decreased SRI expression at the mRNA level in Huh-7 and Hep3B cells (**p < 0.01,*p < 0.05). **E**–**H** Upregulated ANXA7 increased SRI expression at the protein level and promoted EMT, whereas downregulated ANXA7 decreased SRI expression at the protein level and inhibited EMT in Huh-7 and Hep3B cells (**p < 0.01,*p < 0.05). **I**–**J** IF was used to detect E-cadherin and cytokeratin. **K**, **L** IF assay was used to detect the N-cadherin and Vimentin in Huh-7/Hep3B cells.

It was found that when the expression of ANXA7 was upregulated, the mRNA and protein levels of SRI also increased significantly, Whereas when the ANXA7 was downregulated, the expression of SRI decreased too (Fig. 3A–H, Fig. S1B). At the same time, ANXA7 upregulation reduced the expression of epithelial markers (E-cadherin and cytokeratin) and promoted the interstitial markers (N-cadherin and vimentin) at the protein level, conversely, downregulation of ANXA7 inhibited EMT (Fig. 3E–H). There were no statistically significant differences between the control group and the negative control group. Similarly, cellular immunofluorescence experiments further confirmed the above results (Fig. 3I–L).

### ANXA7 affects the aggressive biological behaviors in vitro

**Fig. 4:**
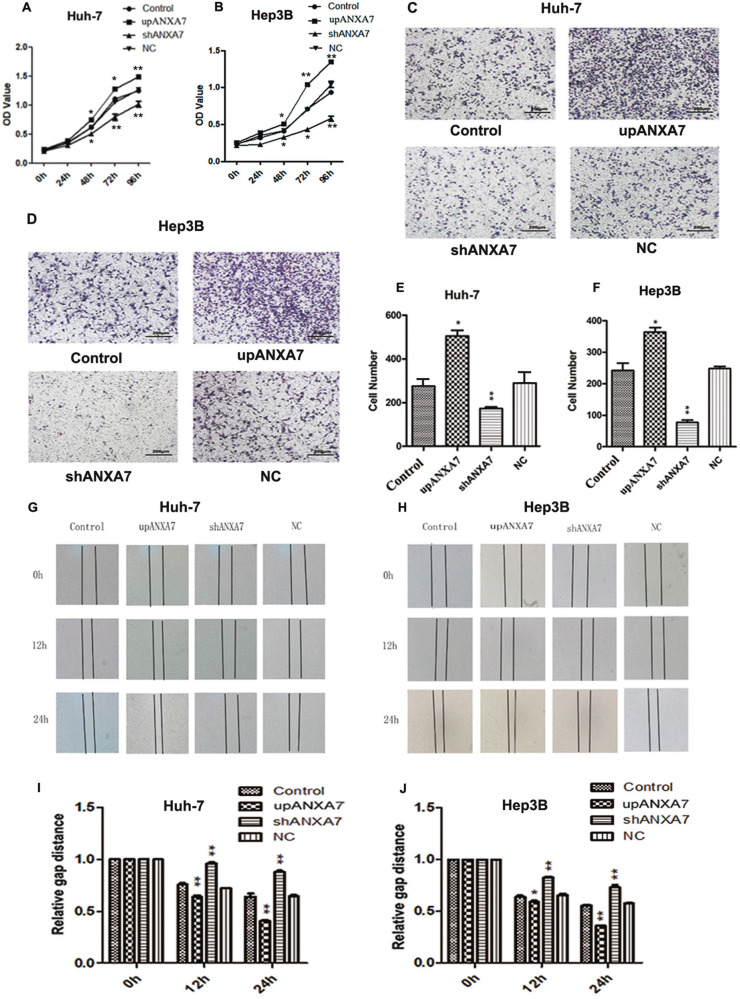
The expression of ANXA7 affects the proliferation, invasion, and migration of HCC cells. **A**, **B** CCK8 assays showed that ANXA7 upregulation promoted the proliferation, whereas ANXA7 downregulation suppressed the proliferation of Huh-7/Hep3B cells (*p < 0.05). **C**–**F** Transwell invasion assays showed that ANXA7 upregulation promoted invasion, whereas ANXA7 downregulation inhibited invasion of Huh-7/Hep3B cells (**p < 0.01, *p < 0.05). **G**–**J** Representative images of wound-healing assays showed ANXA7 upregulation promoted the migration of HCC cells, whereas ANXA7 downregulation inhibited its migration at 0 h,12 h, and 24 h (**p < 0.01, *p < 0.05).

The proliferative capacity of HCC cells was evaluated by CCK8 assay. Our results suggest that the cell proliferation ability of the upregulated ANXA7 group (upANXA7) was significantly higher compared with the control group and negative control group. Furthermore, the cell proliferation ability of the short-hairpin RNA against ANXA7 group (shANXA7) decreased significantly (Fig. 4A, B), No statistical difference was observed between the control group and the negative control group. The results indicated that ANXA7 promotes cell growth.

The invasive capacity of HCC cells was determined by transwell invasion assays. The number of cells transmigrating from within the insert through the membrane in the upANXA7 group was much higher than the control and the negative control group. Although the number of cells that passed through the filter in the shANXA7 group was less than the control and the negative control group (Fig. 4C–F). The control group and the negative control group, however, did not show any significant difference. Altogether these results revealed that ANXA7 promotes the invasive ability of Huh-7 and Hep3B cells.

The migration capacity of HCC cells was determined by wound-healing assays. Upregulation of ANXA7 expression promoted the migration ability of HCC cells and vice versa (Fig. 4G–J). The results showed that ANXA7 promotes cells migration ability. Taking together, ANXA7-SRI interaction promotes EMT and contributes to aggressiveness in HCC in vitro.

### ANXA7 affects the growth, EMT, and metastasis of HCC in vivo

**Fig. 5:**
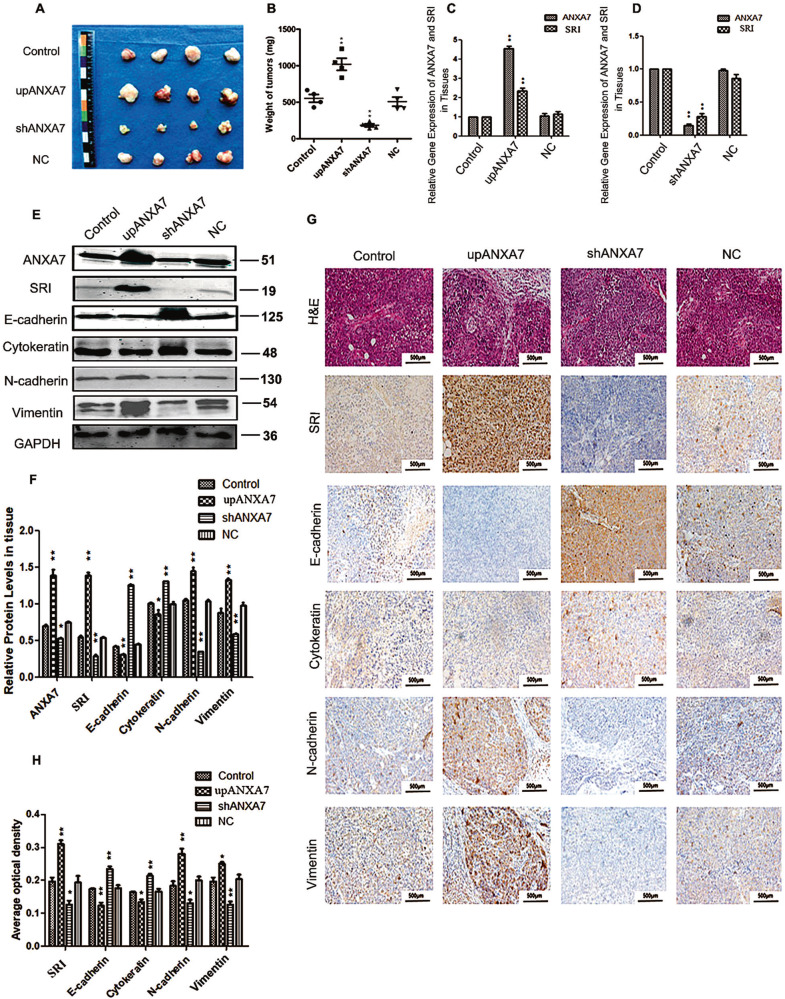
The effect of ANXA7 on tumorigenicity and EMT in vivo. **A**, **B** Images of tumor xenografts and tumor weight (**p < 0.01). **C**, **D** The qRT-PCR analysis of ANXA7 and SRI in tumor xenografts (**p < 0.01). **E**, **F** Western blotting to detect ANXA7, SRI, E-cadherin, Cytokeratin, N-cadherin, and Vimentin in tumor xenografts (**p < 0.01, *p < 0.05). **G**, **H** Immunohistochemical analysis of ANXA7, SRI and EMT indicators in tumor xenografts (**p < 0.01, *p < 0.05).

To further observe the effect of ANXA7 on the growth of HCC cells in vivo, we established a xenograft tumor model, the control cells, upANXA7 cells, shANXA7 cells, and negative control cells in each group subcutaneously. The upANXA7 group showed increased tumor volume and weight, whereas shANXA7 group showed decreased tumor volume and weight (Fig. 5A, B), compared with the control group and the negative control group. We observe the number of the metastatic lymph nodes in upANXA7 group is higher compared with the control group and the negative control group, whereas shANXA7 group is lower by HE staining (Table 1). Extracting the tissue protein of nude mice tumors in each group for qRT-PCR detection (Fig. 5C, D) and western blotting (Fig. 5E, F), we got the same results as in vitro. At the same time, ANXA7 promoted EMT, as well as related markers including E-cadherin, cytokeratin, N-cadherin, and vimentin also change accordingly. Immunohistochemical analysis of tumor tissues in each group further verified the above conclusions (Fig. 5G, H). Consistent with the results obtained in vitro studies, ANXA7 promotes EMT and contributes to aggressive biological behaviors by interacting with SRI.

**Table 1:** Effect of ANXA7 on a metastatic lymph node in vivo.

Group	Number of mice	Number of total lymph nodes	Number of metastatic lymph nodes
Control	4	16	6
upANXA7	4	18	14*
shANXA7	4	18	1*
NC	4	16	6

*indicates statistically significant difference (*p* < 0.05).

### SRI positively regulates the expression of ANXA7 and promotes EMT

To further study the expression of ANXA7 and its influence on EMT after SRI expression changed, we established SRI upexpression and down-expression in HCC cell lines (Huh-7 and Hep3B) (Fig. S2A).

**Fig. 6:**
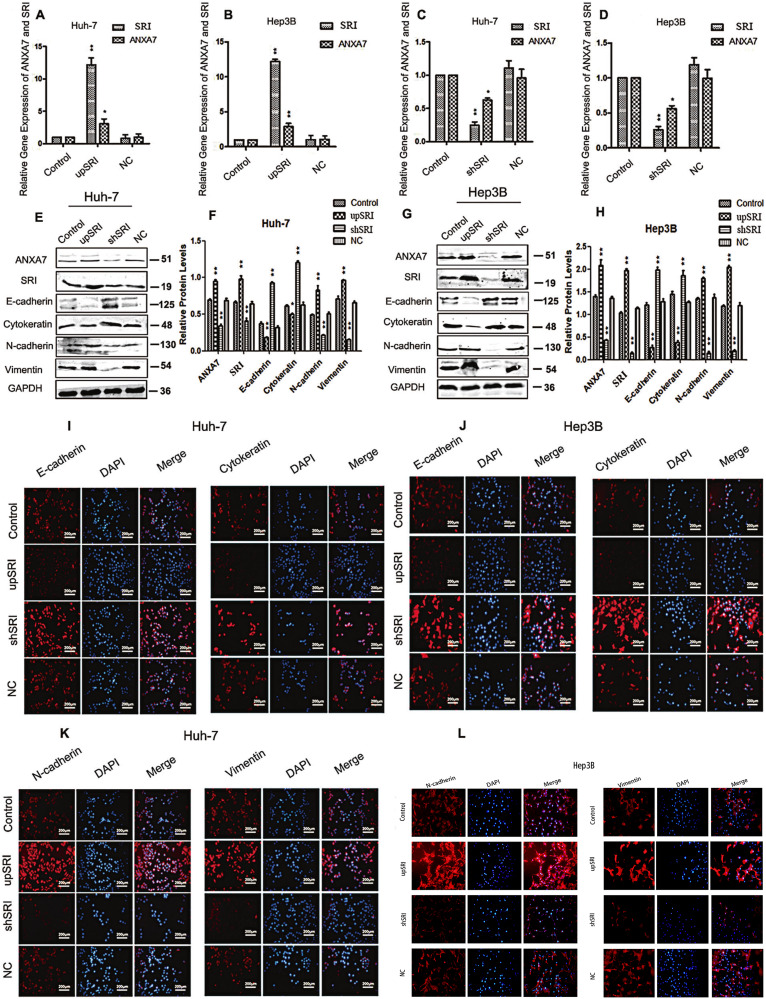
The expression of ANXA7 and EMT indicators after upregulating and downregulating SRI. **A**–**D** When SRI is upregulated, the expression of ANXA7 increased significantly, while when SRI is downregulated, the expression of ANXA7 also decreased in Huh-7 and Hep3B cells by qRT-PCR (**p < 0.01, *p < 0.05). **E**–**H** When SRI is upregulated, the expression of ANXA7 increased, and promoted EMT, whereas when SRI is downregulated, the expression of ANXA7 also decreased, and inhibited EMT in Huh-7 and Hep3B cells by western blotting (**p < 0.01, *p < 0.05). **I**, **J** IF was used to detect the E-cadherin and Cytokeratin after upregulating and downregulating SRI. **K**, **L** IF was used to detect the N-cadherin and Vimentin in Huh-7/Hep3B cells after upregulating and downregulating SRI.

When SRI expression was upregulated in Huh-7 and Hep3B cells, ANXA7 expression increased and promoted the development of EMT. On the contrary, when the expression of SRI is downregulated, the expression of ANXA7 and EMT-related indicators were contrary to the former situation (Fig. 6A–H, Fig. S2B). Meanwhile, cellular immunofluorescence experiments further verified the above conclusions (Fig. 6I–L).

### SRI affects the aggressive biological behaviors in HCC cells

**Fig. 7:**
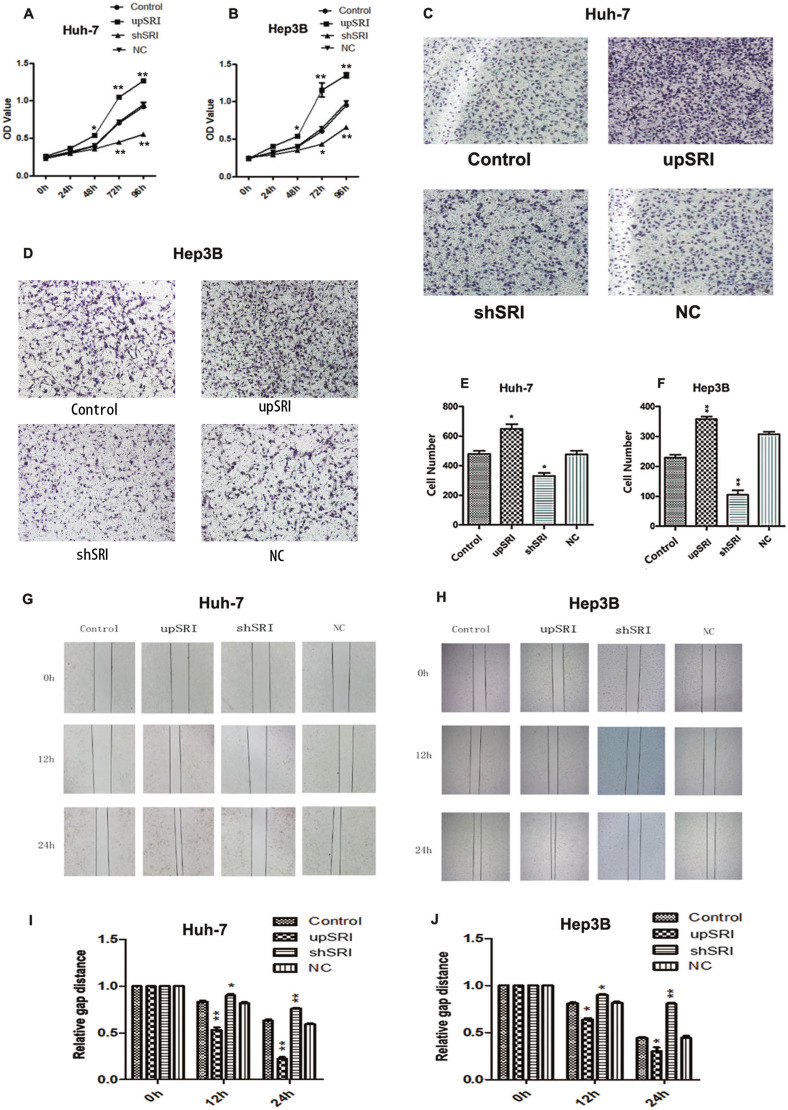
SRI dysregulation affects the invasion, proliferation, and migration of HCC cells. **A**, **B** CCK8 assay showed that SRI upregulation promoted proliferation, whereas SRI downregulation suppressed proliferation of Huh-7/Hep3B cells (*p < 0.05). **C**–**F** Transwell invasion assays showed that SRI upregulation promoted invasion, whereas SRI downregulation inhibited invasion of Huh-7/Hep3B cells (**p < 0.01,*p < 0.05). **G**–**J** Representative images of migration assays of Huh-7 and Hep3B cells showed SRI upregulation promoted the migration, otherwise, SRI downregulation inhibited its migration at 0 h,12 h, and 24 h (**p < 0.01, *p < 0.05).

The CCK8 assays proved that increasing the expression of SRI promoted the proliferation ability of Huh-7 and Hep3B cells, whereas the opposite inhibited the proliferation capacity of HCC cells (Fig. 7A, B).

Transwell invasion assays showed that increasing the expression of SRI enhanced the invasiveness of Huh-7 and Hep3B cells. The reverse occurred upon decreasing SRI expression compared with the control and negative control group (Fig. 7C–F).

Wound-healing assays demonstrated that increasing the expression of SRI improved the migration ability of Huh-7 and Hep3B cells. Meanwhile, downregulation of SRI inhibited the migration ability. There was no significant difference in the control and negative control group in the above biological behaviors of the cells (Fig. 7G–J).

## Discussion

Accumulated evidence indicates that dysregulation of ANXA7 is associated with the occurrence, invasion, metastasis, and progression of a variety of cancers, but ANXA7 plays different roles in different tumors. ANXA7 might act as a tumor suppresser gene in glioblastoma multiforme [40], glioblastoma [41, 42], melanoma [43], prostate cancer [44–47] and breast cancer [48]; ANXA7 might specifically function as a tumor promoter gene in HCC [49, 50], gastric cancer [51, 52], nasopharyngeal carcinoma [53], and colorectal cancer [54]. The oncogenic properties of ANXA7 have been verified in different lymphatic node metastasis of mouse HCC in our previous studies [55, 56]. However, the molecular mechanisms of ANXA7 underlying HCC metastasis are largely unclear. SRI is widely distributed in the heart and skeletal muscles and is highly expressed in HCC [57], gastric cancer [58], and other cancers [59]. Moreover, SRI is closely related to multidrug resistance and interacts with other proteins to participate in the progression and treatment of tumors. Our previous studies demonstrated that SRI is involved in the metastasis of HCC [34]. Similarly, the molecular mechanisms of SRI in HCC are poorly understood.

PPI participates in various kinds of cell functions extensively. Some proteins exert their regulatory effects by activating or inhibiting proteins they interact with. In recent years, the role of PPI in the development of tumors is increasingly well-appreciated [60]. The first 31 amino-acid fragments in the amino-terminal of ANXA7 can be combined with SRI, and the carboxyl-terminal of ANXA7 can be combined with calcium ions to form a protein complex to exert the biological effects [37, 59]. We used co-IP to prove that SRI is the interacting partner for ANXA7, and this was further supported by immunofluorescence showed ANXA7 and SRI colocalization which is a strong foundation for their interaction. This is consistent with results from STRING database analysis.

Tumor metastasis is a complex multi-step cascade process, while EMT is a key factor. EMT refers to the biological phenomenon whereby epithelial cells are empowered into motility and become more migratory and invasive, all of which are characteristics of mesenchymal cells. EMT not only participates in the development of the embryo and the tissue repair in the physiological state but also plays a very important role in the invasion and metastasis of tumors in the pathological state. Cells that undergo EMT exhibit a decrease in E-cadherin, and Cytokeratin [61, 62] as well as an increase in N-cadherin, vimentin [63, 64]. Studying the related molecules involved in the EMT process and the specific mechanism of their functions will help to improve the treatment and prognosis of tumor patients.

In Huh-7 and Hep3B cells, when the ANXA7 was upregulated, the SRI also increased, at the same time, the epithelial phenotype markers (E-cadherin, cytokeratin) decreased, and the interstitial phenotype markers (N-cadherin, vimentin) increased which promoted the development of EMT. Then invasion, proliferation, and migration abilities of HCC cells were enhanced. Downregulating the ANXA7, the expression of SRI also decreased, whereas the EMT markers also changed in the opposite direction, and then metastasis abilities of HCC cells were inhibited simultaneously. The same conclusions were also verified in vivo by conducting a mouse xenograft model. In order to enhance the explanatory power of the experiment, we also change the expression of SRI, and got the same conclusion as the previous part of the experiment. While further mechanisms of the interaction between ANXA7 and SRI affecting the aggressiveness of HCC still need to be clarified.

**Fig. 8:**
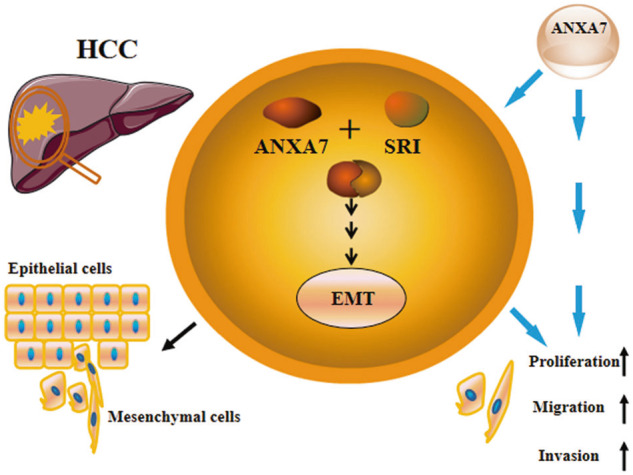
Proposed schematic diagram about the mechanism of ANXA7 in HCC. Interaction between ANXA7 and SRI affects EMT progress and contributes to the aggressiveness in HCC.

We have uncovered ANXA7 and SRI are proteins that interact with each other. They work together to promote EMT and contribute to the proliferation, invasion, and migration of HCC (Fig. 8). Our current work provides new insights into the underlying mechanism of metastasis and represents a potential therapeutic target for HCC.

## Supplementary information

Supply figure legends

Figure S1

Figure S2

## Data Availability

All the relevant data during the current study are available from the corresponding author on reasonable request.
