# Correction: AnnexinA7 promotes epithelial–mesenchymal transition by interacting with Sorcin and contributes to aggressiveness in hepatocellular carcinoma

**DOI:** 10.1038/s41419-024-07184-6

**Published:** 2024-11-29

**Authors:** Fei Ling, Huan Zhang, Yunliang Sun, Jinyi Meng, Jaceline Gislaine Pires Sanches, He Huang, Qingqing Zhang, Xiao Yu, Bo Wang, Li Hou, Jun Zhang

**Affiliations:** 1 Department of Pathology and Forensics, College of Basic Medical Sciences, Dalian Medical University, Dalian, 116044 China; 2 Department of Pathology, Dalian Municipal Central Hospital affiliated with Dalian Medical University, Dalian, 116033 China

Correction to: *Cell Death and Disease* 10.1038/s41419-021-04287-2, published online 29 October 2021

The author notes that the original version of this article contains inaccuracies in Figs. 6L and 7D.

Due to negligence in image layout, the author mistakenly pasted the image of the NC group for the control group in Fig. 6L (Vimentin). The author mistakenly pasted the transwell image of Fig. 4D (upANXA7 group) for Fig. 7D (upSRI group). The correct Figs. 6L and 7D are provided below. The modifications to Figs. 6L and 7D do not affect the results or conclusions reported in the paper.

The author apologizes for any inconvenience caused.

(Original data) Fig. 6L control group -DAPI
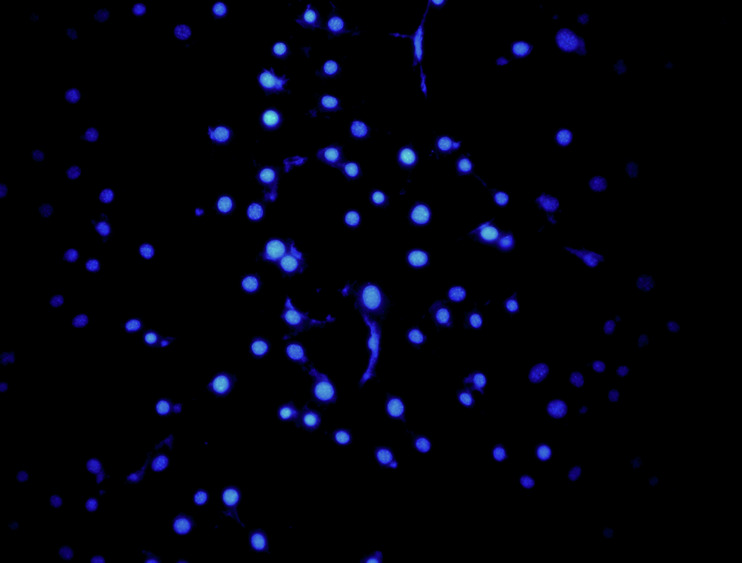


(Original data) Fig. 6L control group -merge
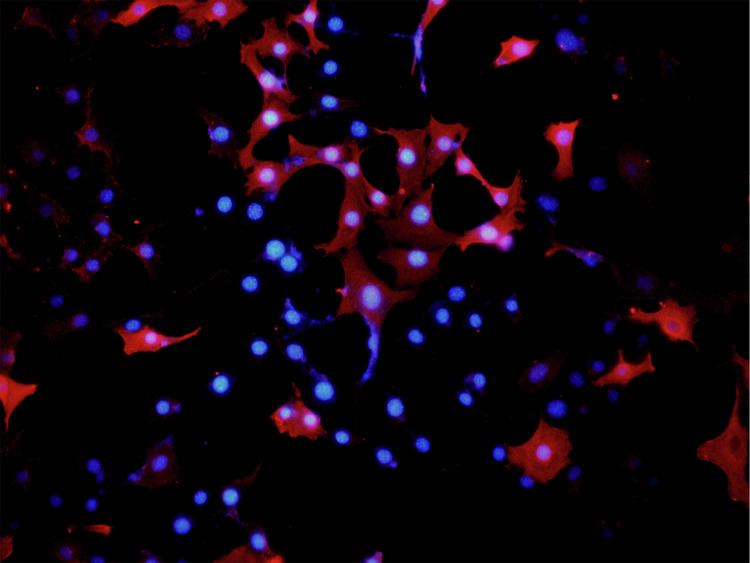


(Original data) Fig. 6L control group -vimentin
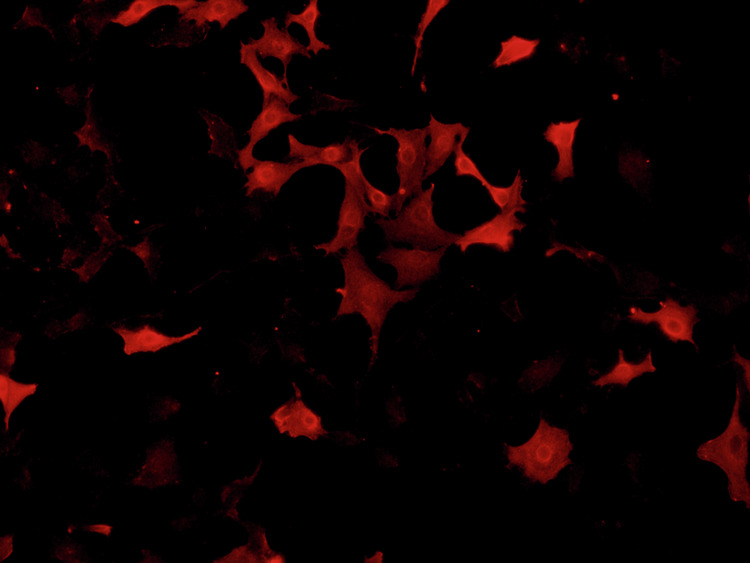


(Original data) Fig. 7D (upSRI group)
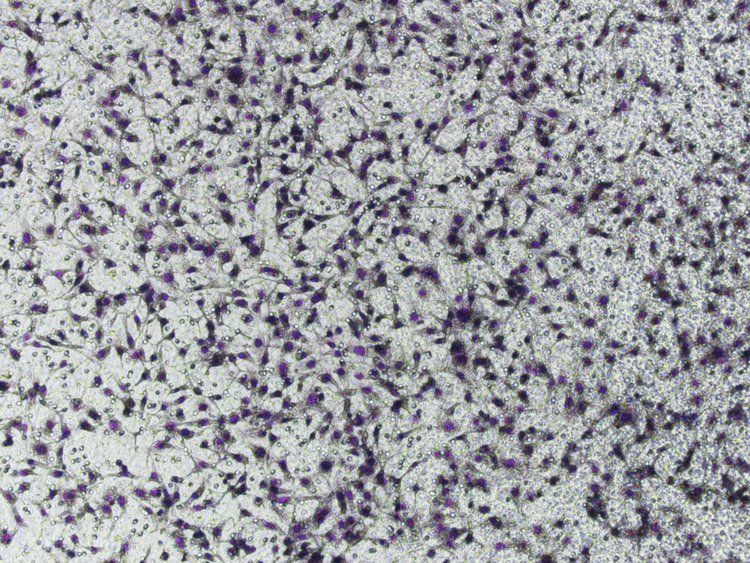


Amended Fig. 6L
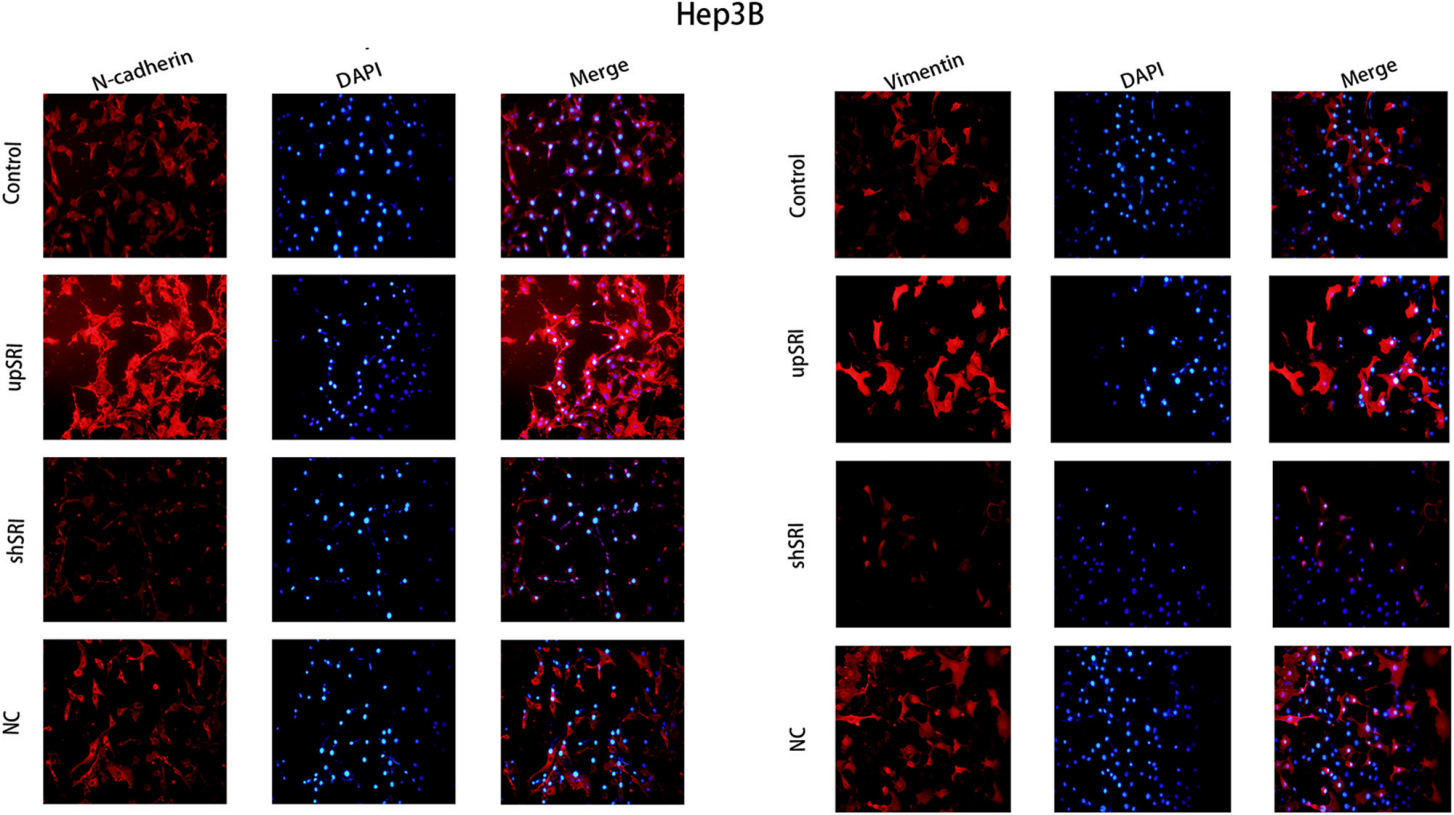


Amended Fig. 7D
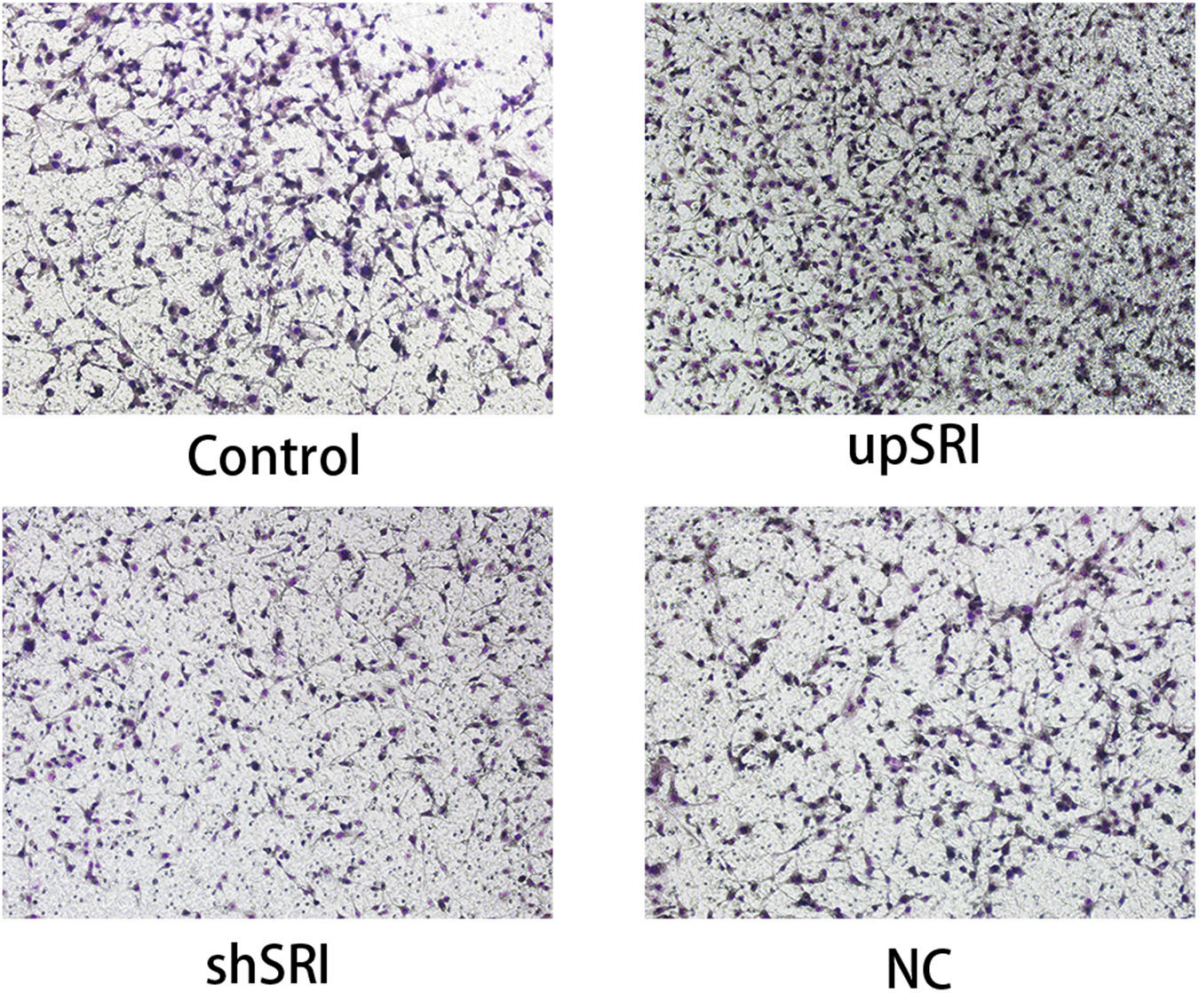


The original article has been corrected.

