# Supplementary material for: AnnexinA7 promotes epithelial–mesenchymal transition by interacting with Sorcin and contributes to aggressiveness in hepatocellular carcinoma
Source: Cell Death Dis. 2021 Oct 29;12(11):1018. doi: 10.1038/s41419-021-04287-2 (PMC8556303; doi:10.1038/s41419-021-04287-2)
Supplement: Supplementary file 1 — Supply figure legends [file 41419_2021_4287_MOESM1_ESM.docx]

**Supply figure legends：**

**Fig.****S1 ANXA7 dysregulation cells were successfully constructed， and IF analysis of ANXA7 and SRI in Huh-7 and Hep3B cells.** (A) ANXA7 up-regulated and ANXA7 down-regulated cells were successfully constructed in Huh-7 and Hep3B cells. **(B)** Up-regulated and down-regulated ANXA7 in Huh-7 / Hep3B cells, SRI was detected by IF.

**Fig.S2** **SRI dysregulation cells were successfully constructed，and IF analysis of** **ANXA7 and** **SRI in** **Huh-7 and Hep3B cells.** (A) SRI up-regulated and SRI down-regulated cells were successfully constructed in Huh-7 and Hep3B cells. (B) Up-regulated and down-regulated SRI in Huh-7 / Hep3B cells, ANXA7 was detected by IF.
