# Supplementary figures and images for: AnnexinA7 promotes epithelial–mesenchymal transition by interacting with Sorcin and contributes to aggressiveness in hepatocellular carcinoma
Source: Cell Death Dis. 2021 Oct 29;12(11):1018. doi: 10.1038/s41419-021-04287-2 (PMC8556303; doi:10.1038/s41419-021-04287-2)

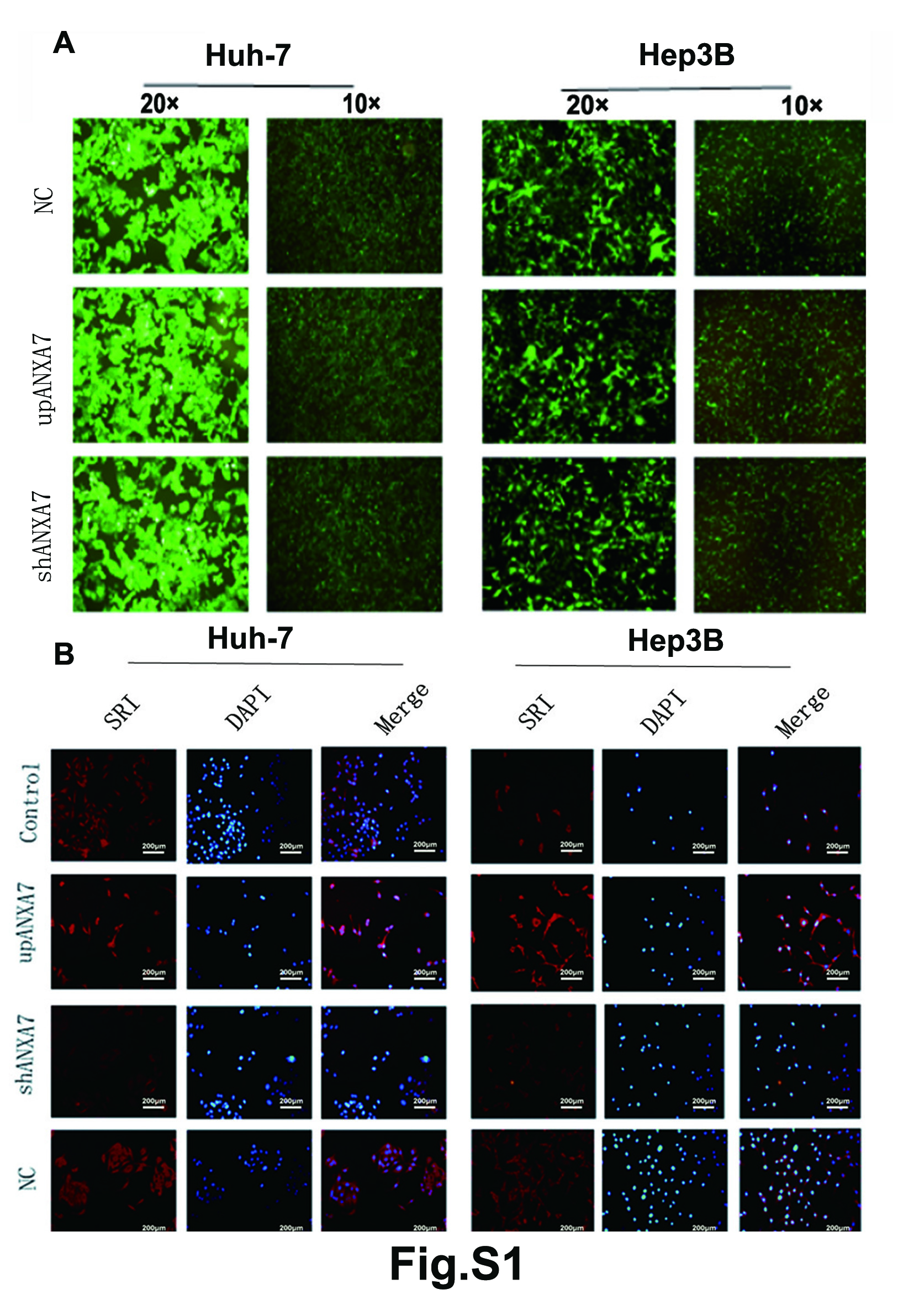

Supplement: Supplementary file 2 — Figure S1 [file 41419_2021_4287_MOESM2_ESM.tif]

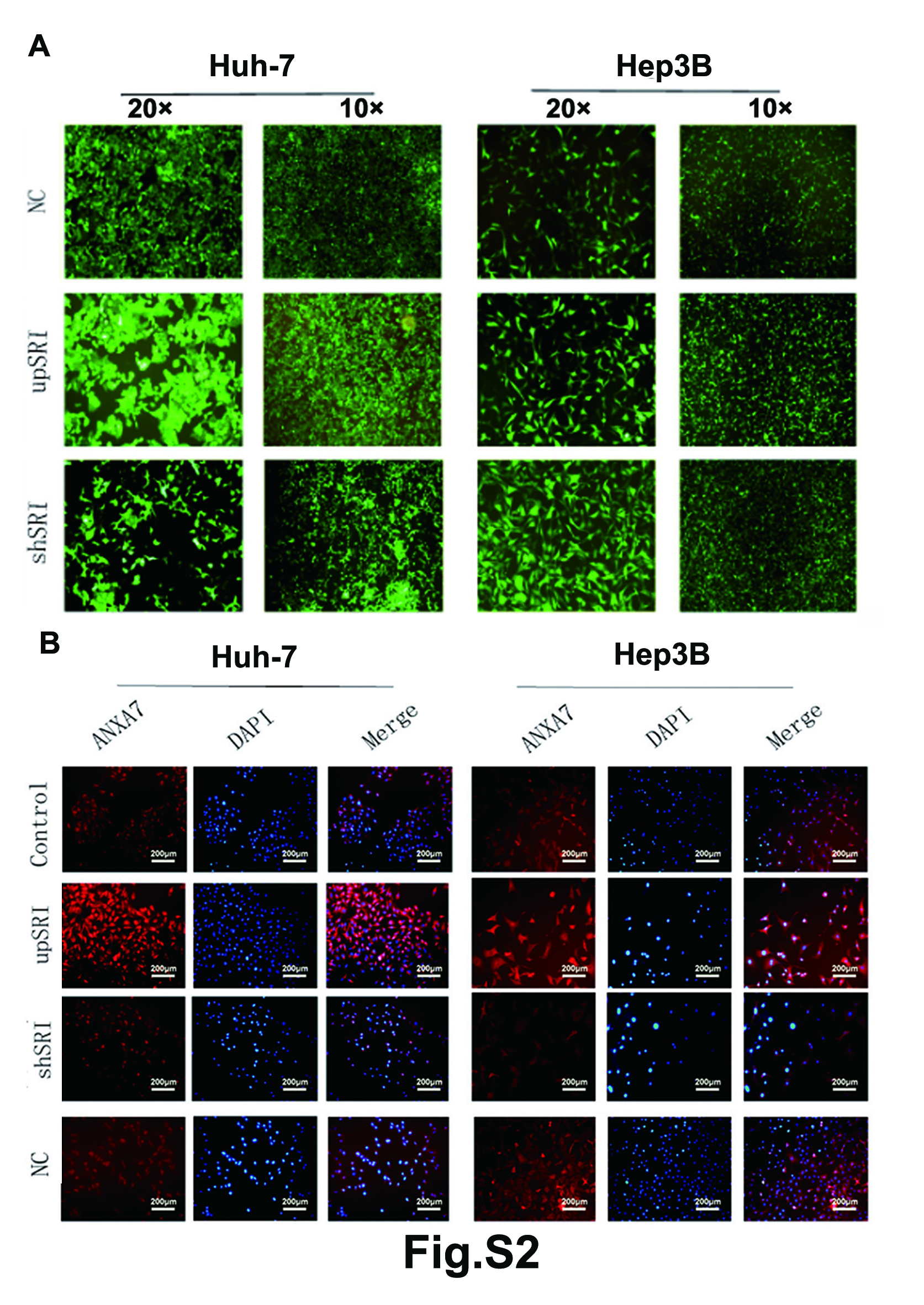

Supplement: Supplementary file 3 — Figure S2 [file 41419_2021_4287_MOESM3_ESM.tif]
